# Allergen‐specific immunotherapy for patients with atopic dermatitis sensitized to animal dander

**DOI:** 10.1002/iid3.291

**Published:** 2020-03-12

**Authors:** Howard Chu, Kyung Hee Park, Su Min Kim, Jae‐Hyun Lee, Jung‐Won Park, Kwang Hoon Lee, Chang Ook Park

**Affiliations:** ^1^ Department of Dermatology, Cutaneous Biology Research Institute Yonsei University College of Medicine Seoul Korea; ^2^ Division of Allergy, Department of Internal Medicine Yonsei University College of Medicine Seoul Korea; ^3^ Institute of Allergy Yonsei University College of Medicine Seoul Korea

**Keywords:** allergen‐specific immunotherapy, animal dander, atopic dermatitis

## Abstract

**Introduction:**

Atopic dermatitis (AD) is a chronic inflammatory skin disease, and AD patients are commonly sensitized to house dust mite (HDM). Of the several treatment options available, allergen‐specific immunotherapy (AIT) has been recognized as an effective treatment modality that is directed toward the immunoglobulin E (IgE)‐mediated nature of AD, and subcutaneous administration using HDM is most commonly used for AIT in AD. For patients sensitized to animal (dog or cat) dander, the treatment may not be easy, especially when avoiding the allergen is not possible.

**Methods:**

This study enrolled patients with AD who were sensitized to cat and/or dog dander and underwent AIT (n = 19). Patients’ medical information was obtained, including past treatment history, treatment duration of AIT, and the progress of treatment. Also, the specific IgE levels and IgG4 levels were measured before and after AIT.

**Results:**

A total of 19 patients with AD underwent AIT using cat and/or dog dander. The patients consisted of 4 males and 15 females with an average age of 31.74 ± 9.71. Only two patients had AD only, and the other 17 patients had one or more concomitant allergic diseases, such as allergic rhinitis, allergic asthma, or allergic conjunctivitis. Seven patients were not sensitized to HDMs and only sensitized to cat and/or dog dander. The duration of AIT ranged from 2 to 58 months. The symptoms of 17 patients were well‐controlled, requiring only topical treatment and/or oral antihistamines. One patient required systemic cyclosporine, but only of low dose (25 mg/day). The specific IgE levels were decreased (*P* = .005) and IgG4 levels showed the tendency of increasing after AIT. No adverse events were observed in these patients.

**Conclusion:**

Although a larger number of patients for a longer follow‐up period are needed to precisely assess the treatment efficacy, AIT using cat and/or dog dander may be an effective treatment option for AD patients, especially for severe AD patients with other respiratory allergic comorbidities who cannot completely avoid the exposure to animal dander.

## Graphical abstract

As the sensitization to animal dander, predominantly cat and dog dander, constitutes a significant portion in patients with atopic dermatitis (AD), treatment modalities directed toward the desensitization of these allergens should also be available. While allergen‐specific immunotherapy using cat and/or dog dander is performed in patients with respiratory allergic diseases, there has been no report for AD, and in this study, we found that it was effective in AD as well.

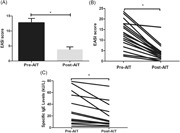

**Abbreviations**
AD: atopic dermatitis
AIT: allergen‐specific immunotherapy
EASI: eczema area and severity index
HDM: house dust mite
Ig: immunoglobulin
IGA: investigator global assessment

Atopic dermatitis (AD) is a chronic inflammatory skin disorder characterized by relapsing eczematous skin lesions with marked pruritus. It is a multifactorial disorder that results from genetic susceptibility with barrier disruption and immune dysregulation, in addition to environmental triggers.^1^ A major portion of the patients is sensitized to allergens, most commonly to house dust mite (HDM), in which serum total immunoglobulin E (IgE) and specific IgE to certain allergens are found to be increased.^2^ For these sensitized patients, among various available treatment options, allergen‐specific immunotherapy (AIT) is the treatment that is directed towards the IgE‐mediated nature of the disease.^3^

AIT has been used in allergic diseases, more predominantly in allergic rhinitis and allergic asthma, for the past decades.^3^ Although the evidence of its efficacy may not be as concrete in AD, studies suggesting the effectiveness in AD is steadily increasing.^4^ In comparison to AIT in other allergic diseases that use various kinds of allergens (eg, pollen, HDM, animal danders), the effectiveness of AIT in AD has been established in patients sensitized to HDM.^5^ Although HDM is predominant allergen sensitized in AD patients, some patients are concomitantly sensitized to other allergens or may be sensitized to allergens other than HDM in some cases.^6^ Among the various allergens, animal dander, particularly cat and dog dander, can be found to be sensitized in a significant proportion of patients, and has become an issue as the number of pet owners is steadily increasing.^7^ Also, studies have found that AD patients sensitized to animal dander have a greater likelihood to develop other respiratory allergic diseases.^6^ AIT using cat and/or dog dander has been used for the treatment of allergic asthma or allergic rhinitis,^8^, ^9^ whereas the use of cat and/or dog extract for AIT in AD patients has not been reported.^10^

Among patients diagnosed with AD according to the diagnostic criteria of Hanifin and Rajka,^11^ those sensitized to cat and/or dog dander, regardless of sensitization to HDM, and underwent AIT were enrolled. The patients underwent AIT using Hollister‐Stier aqueous extracts (Hollister‐Stier, Spokane, WA) if (a) they asserted that their symptoms aggravate when in contact with cat and/or dog, (b) greater than equal to 3.5 kU_A_/L (class 3) serum specific IgE levels to cat or dog dander confirmed by CAP immunoassay, and (c) inadequately controlled symptoms with topical therapies. This study has been approved by the Institutional Review Board of Severance Hospital (IRB no. 4–2018‐0334). Table 1 shows the demographics, comorbid allergic diseases, treatment modality, duration of the treatment, and the treatment progresses of each patient. There were a total of 19 patients, which consisted of 4 males and 15 females with an average age of 31.74 ± 9.71 and an age range between 17 and 55. Seventeen patients had one or more concomitant allergic diseases, such as allergic rhinitis, allergic asthma, or allergic conjunctivitis, and the remaining two patients had only AD. Patients were initially assessed for the allergens sensitized by CAP immunoassay, and their serum specific IgE levels and IgG4 levels were assessed. Seven patients were not sensitized to either *Dermatophagoides farinae* or *Dermatophagoides pteronyssinus* and only sensitized to cat and/or dog dander and the rest of the patients were sensitized to both HDM and cat/dog dander. The duration of AIT that the patients underwent ranged from 2 to 58 months. Of the 19 patients, one patient discontinued AIT due to exacerbation of AD. For the remaining subjects, the symptoms of 17 patients were well‐controlled, requiring only topical treatment and/or oral antihistamines (Figure 1). One patient required systemic cyclosporine, but only of minimal dosage (25 mg/day). Clinical severity of AD was assessed by Investigator Global Assessment (IGA) and Eczema Area and Severity Index (EASI) and a significant reduction of IGA scores and EASI scores were observed after AIT by the Wilcoxon signed‐rank test (IGA: n = 19; *P* = .0009; EASI: n = 19; *P* = .0017), (Figures 2).

**Table 1:** Demographics, comorbid allergic diseases, treatment modality, duration of the treatment, and the treatment progresses of atopic dermatitis patients who underwent allergen‐specific immunotherapy with the cat and/or dog dander

Patient number	Age	Sex	Comorbid allergic diseases	Allergens used for AIT	Type of treatment	Treatment duration, mo	Treatment progress
1	41	M	AA, AR	d1,d2,e1,e5	Cluster	17	Well‐controlled with topicals and antihistamines
2	33	F	AR, AC, AA	e1	Rush	16	Well‐controlled with topicals and antihistamines
3	29	F	AD only	e5	Rush	16	Requires low dose cyclosporine (25 mg/day)
4	19	F	AA, AR	d1, d2, e1, e5	Cluster	14	Well‐controlled with topicals only
5	26	F	AA, AR	d1, d2, e1	Cluster	29	Well‐controlled with topicals and antihistamines
6	27	M	AR	e1, e5	Rush	14	Well‐controlled with topicals
7	38	M	AR	d1,d2,e1,e5	Rush	12	Well‐controlled with only AIT
8	29	F	AR	d1,d2,e1,e5	Cluster	2	Discontinuation due to acute exacerbation
9	26	F	AR	d1, d2, e1, e5	Rush	22	Well‐controlled with topicals only
10	25	F	AD only	d1, d2, e5	Cluster	9	Well‐controlled with only AIT
11	17	M	AR	d1,d2,e1	Cluster	16	Well‐controlled with only AIT
12	28	F	AR, AC, AA	d1,d2,e1,e5	Cluster	6	Well‐controlled with only AIT
13	55	F	AR	e5	Cluster	36	Well‐controlled with topicals and antihistamines
14	49	F	AR, AA	e1,e5	Cluster	21	Well‐controlled with topicals and antihistamines
15	31	F	AR, AA	e5	Rush	29	Well‐controlled with only AIT
16	34	F	AR, AA	d1, d2, e1, e5	Cluster	58	Well‐controlled with topicals and antihistamines
17	39	F	AR	e1, e5	Cluster	39	Well‐controlled with topicals and antihistamines
18	21	F	AR, AA	d1, d2, e1	Rush	8	Well‐controlled with topicals and antihistamines
19	36	F	AR	d1, d2, e5	Rush	9	Well‐controlled with topicals and antihistamines

Abbreviations: AA, allergic asthma; AD, atopic dermatitis; AIT, allergen‐specific immunotherapy; AR, allergic rhinitis; d1, *Dermatophagoides pteronyssinus*; d2, *Dermatophagoides farinae*; e1, cat dander; e5, dog dander.

**Figure 1:**
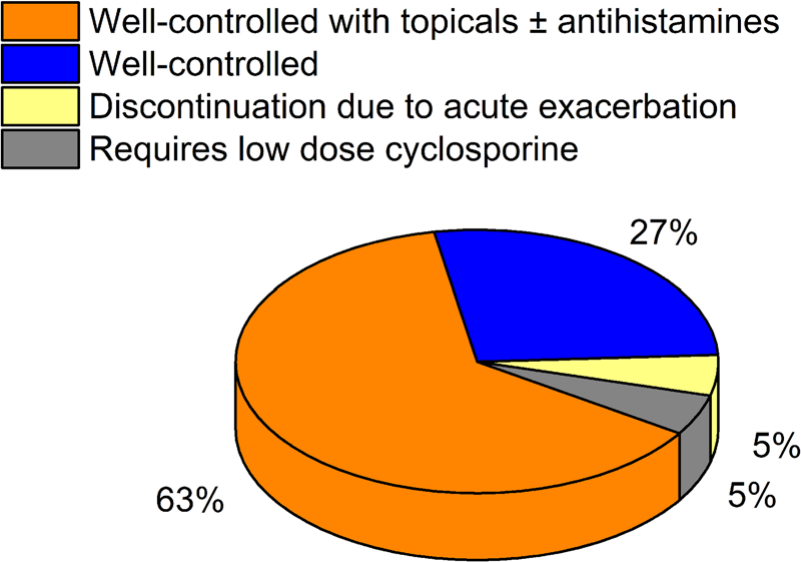
The treatment progress of the patients after AIT. AIT, allergen‐specific immunotherapy

**Figure 2:**
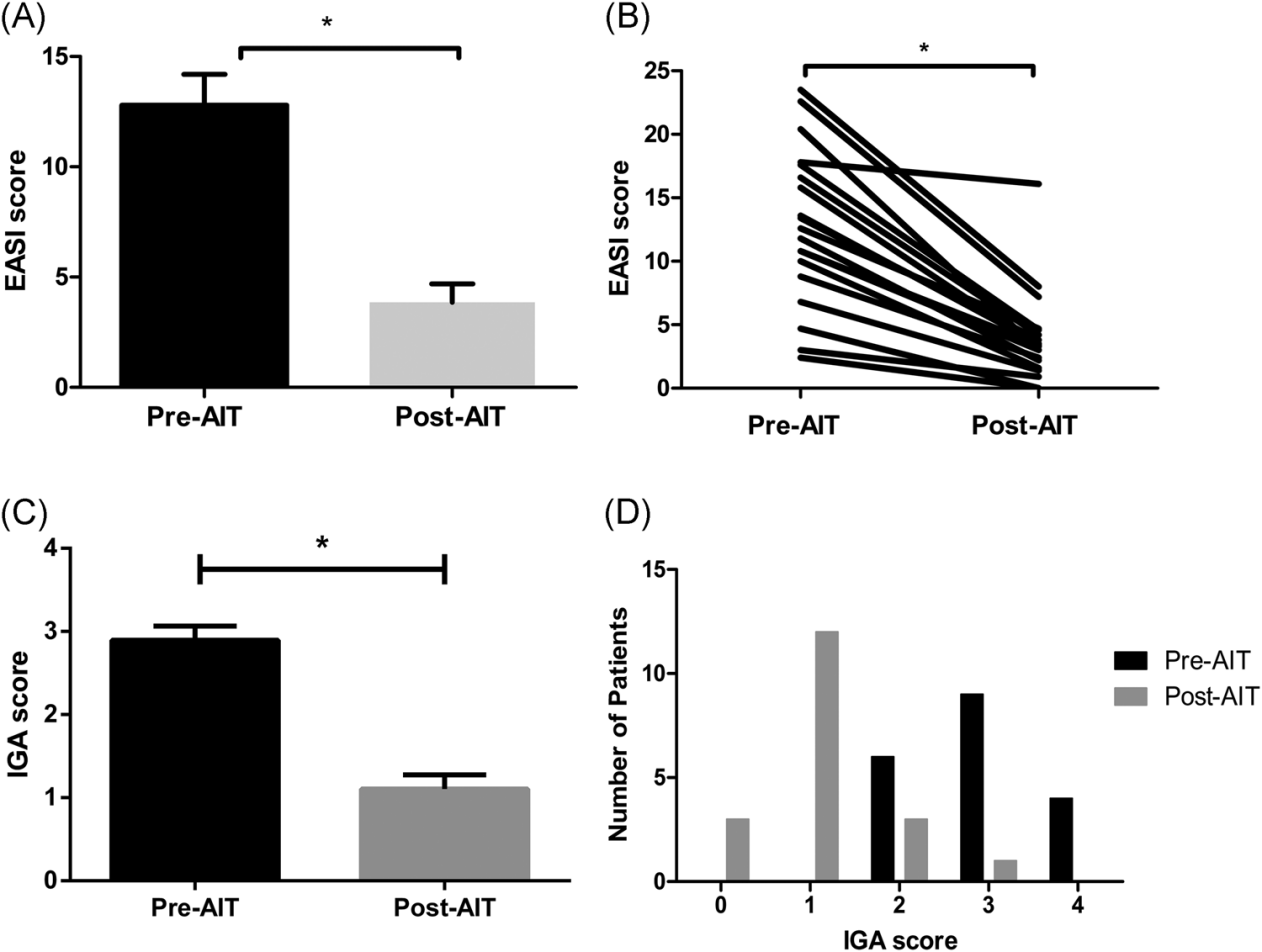
A, The change of the eczema area and severity index (EASI) scores before (pre‐AIT) and after (post‐AIT) allergen‐specific immunotherapy with animal dander. B, EASI scores from 19 patients were significantly reduced. C, The investigator global assessment (IGA) score was significantly reduced post‐AIT and D, The patients had higher IGA scores pre‐AIT, whereas the scores post‐AIT were distributed in lower numbers (n = 19). IGA scores were categorized as follows; 0: clear, 1: almost clear, 2: mild, 3: moderate, 4: severe. *p < 0.05. AIT, allergen‐specific immunotherapy

Specific IgE levels and IgG4 levels were obtained after 1 year in each patient who received AIT for more than 6 months. Specific IgE levels to cat dander and dog dander were decreased after the AIT when compared with the initial levels (Figure 3A), and the difference was statistically significant by the Wilcoxon signed‐rank test (*P* = .005). Specific IgG4 levels to cat and dog dander were found to be increased after the AIT (Figure 3B), but the difference was not significant. No adverse events were observed in the patients, except for the patient whose symptoms had exacerbated during the treatment, requiring the cessation of AIT.

**Figure 3:**
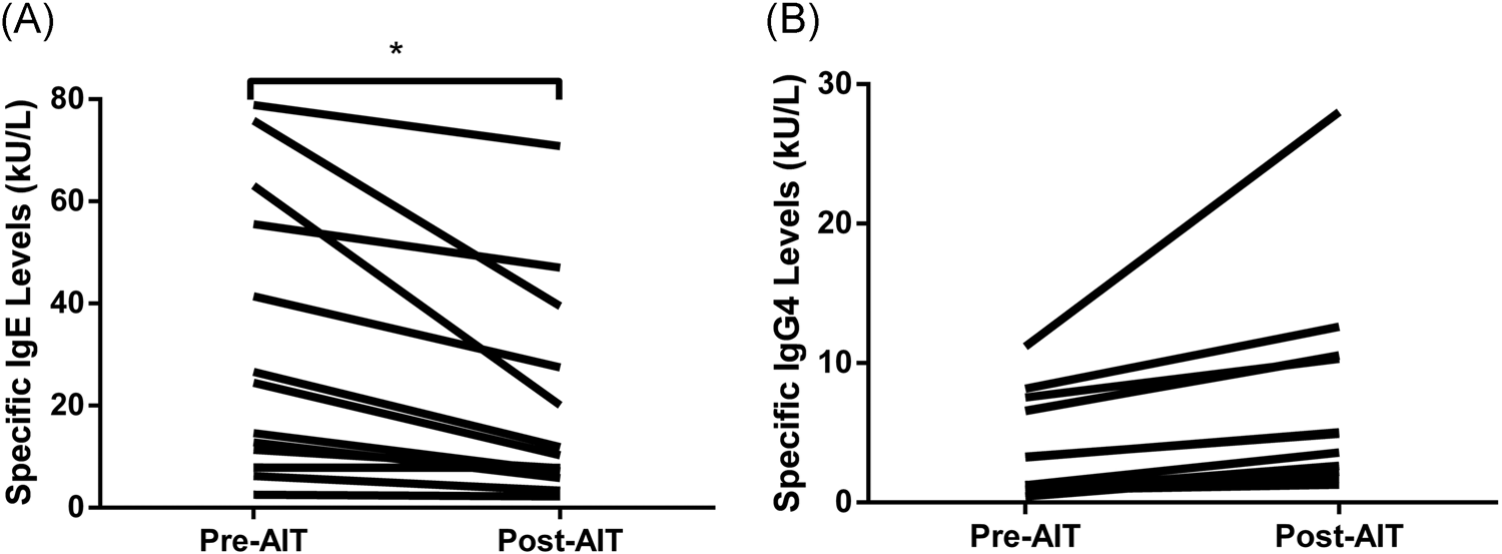
A, Specific immunoglobulin E (IgE) levels, and B, Specific IgG4 levels to animal dander before (pre‐AIT) and after (post‐AIT) allergen‐specific immunotherapy (n = 14). *p < 0.05. AIT, allergen‐specific immunotherapy

Despite the emergence of various novel treatment modalities in AD including dupilumab, there are still some patients who remain irresponsive to these treatments. As the number of pet owners steadily increases^7^ and the complete avoidance of animal dander may not be always feasible, desensitization of the allergens may be the alternative treatment option for some patients. Through this study, we found that patients with AD also could be benefited from AIT with the cat and/or dog dander, as verified in allergic asthma and allergic rhinitis.^8^, ^9^, ^10^ The majority of the patients had shown marked improvement of symptoms, in addition to the decrease in specific IgE levels and increase in specific IgG4 levels. To our knowledge, this is the first study on the result of AIT in patients with AD sensitized to cat and/or dog dander. This study may be limited to the fact that it is an observational study that the sample size may not be so large and included AD patients sensitized to other allergens as well. However, these results may provide insights for new therapeutic options for AD patients sensitized to animal dander, especially for severe AD patients with other respiratory allergic comorbidities who cannot completely avoid exposure to animal dander.
